# Visual and thermal stimuli modulate mosquito-host contact with implications for improving malaria vector control tools

**DOI:** 10.1016/j.isci.2023.108578

**Published:** 2023-11-27

**Authors:** Manuela Carnaghi, Federico Mandelli, Lionel Feugère, Jillian Joiner, Stephen Young, Steven R. Belmain, Richard J. Hopkins, Frances M. Hawkes

**Affiliations:** 1 Department of Agriculture, Health, and Environment, Natural Resources Institute, University of Greenwich at Medway, Chatham, Kent, ME4 4TB, UK; 2 School of Science, University of Greenwich at Medway, Chatham, Kent, ME4 4TB, UK; 3 Gillingham, UK

**Keywords:** Disease, Biological sciences, Entomology, Evolutionary biology

## Abstract

Malaria prevention relies on mosquito control interventions that use insecticides and exploit mosquito behavior. The rise of insecticide resistance and changing transmission dynamics urgently demand vector control innovation. To identify behavioral traits that could be incorporated into such tools, we investigated the flight and landing response of *Anopheles coluzzii* to human-like host cues. We show that landing rate is directly proportional to the surface area of thermal stimulus, whereas close-range orientation is modulated by both thermal and visual inputs. We modeled anopheline eye optics to theorize the distance at which visual targets can be detected under a range of conditions, and experimentally established mosquito preference for landing on larger targets, although landing density is greater on small targets. Target orientation does not affect landing rate; however, vertical targets can be resolved at greater distance than horizontal targets of the same size. Mosquito traps for vector control could be significantly enhanced by incorporating these features.

## Highlights

• Development of an insect eye model that estimates resolvable distances of targets
• Area of thermal cue influences landing in *Anopheles* mosquitoes
• Size, but not spatial orientation, influences target attractiveness and efficiency
• Visual and thermal cues drive close-range attraction; thermal cues drive landing

## Graphical abstract

**Figure:**
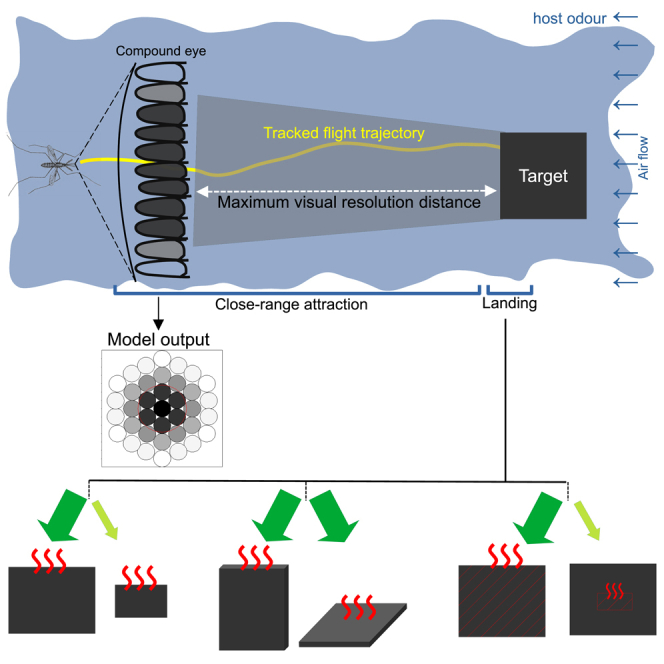

## Introduction

Malaria, a parasitic disease transmitted by mosquitoes, affects yearly over 200 million people worldwide.^1^ Current malaria prevention relies on tools that control the vectors, which are solely mosquitoes of the *Anopheles* genus, and the use of preventive medicines in target human populations.^2^ Dominant mosquito control techniques rely on insecticides to kill mosquitoes, and with insecticide-resistant traits spreading at an alarming rate,^1^ future sustainable malaria control programs need additional control methods.^3^^,^^4^ Novel methods should include non-insecticide approaches^5^ and should also aim to reduce outdoor malaria transmission.^6^ Modeling suggests that malaria can be eliminated by 2040, but only in scenarios where new paradigms in preventing outdoor biting are implemented.^7^ To address the malaria global challenge, various new “lure-and-kill” mosquito traps and targets are in development, aimed at reducing the vector population and ultimately the entomological inoculation rate.^4^^,^^5^^,^^8^^,^^9^^,^^10^^,^^11^ Promising reductions in malaria transmission suggest that mass-trapping mosquitoes and “push-pull” systems^12^ could play an important role in diversifying efforts to eliminate the disease in many countries.^5^

Vector control strategies usually exploit aspects of mosquito behavior to kill the insect. Mimicking human characteristics to attract the vector is the basis of a number of mosquito trap designs.^5^^,^^8^^,^^13^^,^^14^^,^^15^^,^^16^^,^^17^ The majority of mosquito traps lure mosquitoes using olfactory cues, which are effective over relatively large distances, and once drawn to the vicinity of a trap, the final stages of close-range orientation are driven by skin odor and visual properties, as well as thermal and humidity gradients.^8^^,^^18^^,^^19^^,^^20^^,^^21^^,^^22^ However, the last stages of host-seeking behavior are poorly understood, and particularly, more research is needed to better understand the role of visual and thermal cues in guiding landing in nocturnal *Anopheles* species, which may differ markedly compared to recent results obtained from diurnal *Aedes aegypti*.^22^^,^^23^ Incorporating the physical stimuli that drive close-range orientation and landing behavior into trap designs may radically improve their effectiveness, but this will require an accurate understanding of how these stimuli interact and affect mosquito behavior.^8^ When considering target development or optimization, it is important to consider all characteristics that influence trap performance. Target size, for example, seems to differentially affect behavioral responses in an adaptive way according to insect taxa. In *Drosophila melanogaster*, deceleration prior to landing is triggered by a combination of absolute target size and its rate of visual expansion over the retina,^24^ but in at least one species of stingless bee, *Scaptotrigona depilis*, very small targets initiate an unusual “accelerated landing” strategy.^25^ The size of the area that delivers a particular cue could also alter the overall mosquito response. While increasing the magnitude of a thermal stimulus increases landing in *Ae. aegypti* mosquitoes until noxious temperatures are reached,^26^ it is unclear how mosquitoes may respond to differences in the surface area of thermal targets. *Anopheles quadrimaculatus* was reported to only alight on small targets when these emit convection currents that are scattered over a much wider area,^27^ thus suggesting that the absolute size of area emitting a thermal stimulus may be highly influential in directing close-range orientation and landing in *Anopheles* mosquitoes. Finally, the position of a target, including its orientation, may also influence behaviors that promote close-range approaches and landing,^28^ as it can affect the distance at which the trap is seen and might indicate whether it is a physical obstacle.^29^ Previous studies that used live human baits reported that the biting sites of *Anopheles* mosquitoes were species-specific and depended on the spatial orientation of the host, with more bites received on the legs and feet in standing hosts, whereas when hosts were lying flat on the ground, bites occurred across body parts closest to the ground.^30^^,^^31^^,^^32^ However, results from these studies present discrepancies and reveal that much is still unclear on the role of host or target spatial orientation alone in driving mosquito behavior. This highlights the need of decoupling target orientation from the confounding factors introduced by using whole human baits.

Understanding how size, thermal area, and orientation influence mosquito landing behavior could have significant implications for designing biorational lures, as has been successfully seen in the control of other vector species. Target size effects on *Anopheles* have not been investigated, but have been tested and effectively manipulated to guide the design of and improve 10-fold the performance of traps for the tsetse flies *Glossina palpalis* and *Glossina fuscipes*, vectors of trypanosomes.^33^^,^^34^^,^^35^^,^^36^ Research findings on the effect of bait orientation on behavior have also been used to great effect to identify vertical, oblong traps as the most attractive for the anthropophagic *G. palpalis*.^36^ There are also a range of practical limitations (e.g., the dependence on a power source, a bulky design, and high cost of production) that hamper application of current target designs at a large scale^3^ and in remote locations.^37^ Effort should be made to improve current trap designs to both facilitate their utilization in the field and find more cost-effective solutions.^38^ Toward this end, we quantified the individual effects of human host-associated stimuli in modulating the landing response of *An. coluzzii*, one of the most significant malaria vectors, on experimental targets.^20^ In the current study, we performed three experiments to characterize the effects of variations in the visual, thermal, and positional properties of targets on flight behavior and landing in *An. coluzzii*, and we present a model for estimating the distance at which visual targets are resolvable by various species of Culicidae, according to anatomical parameters of the eye. Here, we show that large visual targets are both preferred by mosquitoes and induce more frequent landing than small visual targets but catch fewer mosquitoes per unit area. Although horizontal or vertical orientation of targets has no effect on landing, we found that targets with a larger proportion of heated surface area are significantly more attractive. The results highlight promising characteristics for field testing and indicate opportunities to improve the poor capture efficiency of current control and surveillance tools^8^ by increasing mosquito contact with capture or killing mechanisms following olfactory-driven attraction.

## Results

### Mosquitoes prefer larger targets than smaller targets, and visit and land on them more frequently

**Figure 1:**
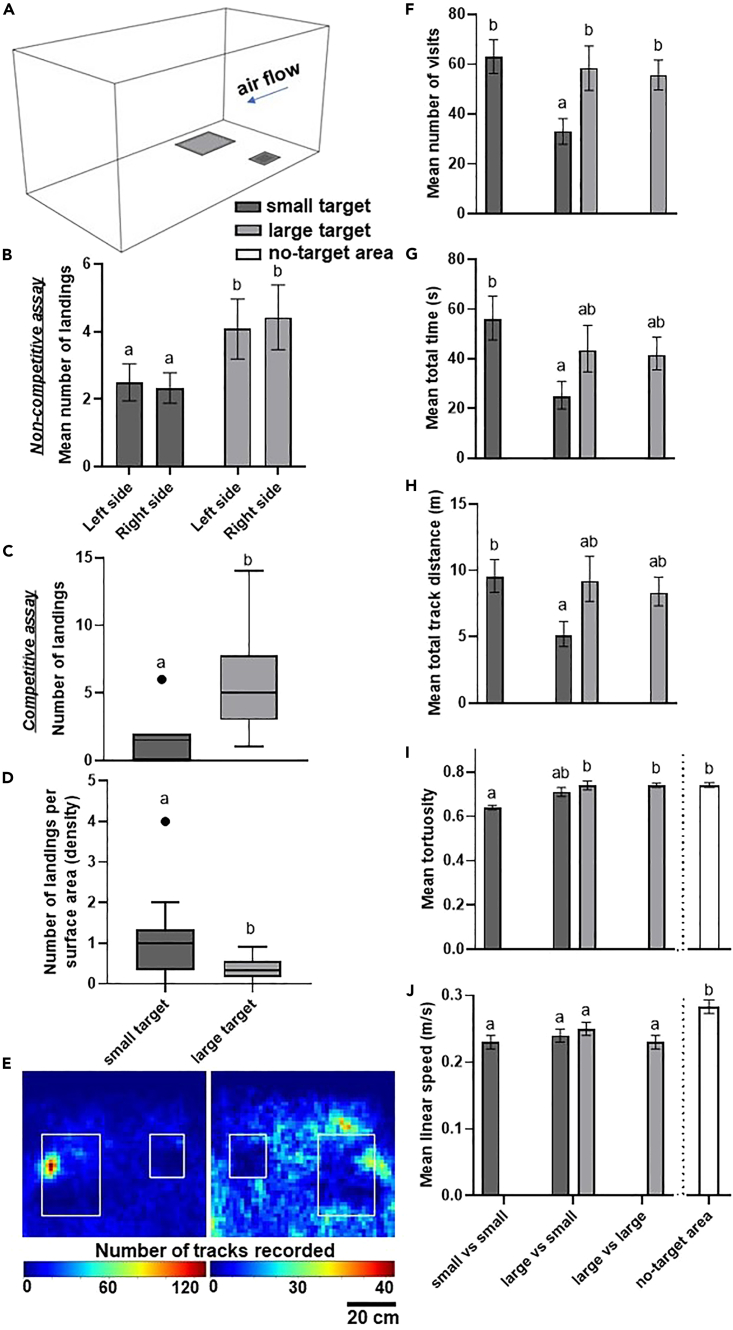
Close-range approach and landing responses of *An.coluzzii* females when presented with same or different size targets (A) Schematic of the wind tunnel with the large (left) and small (right) targets. (B) Mean ± SEM number of mosquitoes that landed on the two target types in non-competitive assays. Bars display raw means while statistical analysis (LMM) was performed on log-transformed data. (C) Box-and-whisker plots show the number of mosquitoes that landed on targets in the competitive assay, data analyzed by Wilcoxon signed-rank test. (D) Box-and-whisker plots show the number of mosquitoes that landed per surface area (i.e., density), data pooled from all assays and analyzed by Wilcoxon signed-rank test. (E) Heat maps of the XY wind tunnel plane showing the density of tracks recorded around the two target types in the competitive assay. White rectangles indicate target position. (F–J) Mean ± SEM of flight track parameters. Data in (F) were analyzed by a GLM followed by Tukey’s post hoc test, while data in (G–J) were analyzed by a two-way ANOVA. For all panels, different lowercase letters indicate significant differences between treatments (p < 0.05).

Female *An. coluzzii* landing responses were explored in a wind tunnel using a three-dimensional tracking system in three assays using combinations of small and large targets. The mosquitoes were simultaneously presented with either a large (30 × 40 cm) and a small target (15 × 20 cm), two large targets, or two small targets (Figure 1A). The number of mosquitoes landing on each target was also recorded. In all assays, mosquitoes were concurrently stimulated with human odors and carbon dioxide to elicit host-seeking.^20^ Both the absolute size of the target and the size of the competing target had a significant effect on the number of mosquitoes that landed on the targets (linear mixed effects model (LMM), ANOVA, F_(1,61)_ = 11.25, p = 0.001; F_(1,61)_ = 5.43, p = 0.02). When presented with two targets of identical size, significantly more mosquitoes landed on large targets, i.e., higher absolute number of landings on the large target (mean ± SEM = 4.7 ± 0.5) than on the small target (2.5 ± 0.4) (LMM, ANOVA, F_(1,65)_ = 10.38, p = 0.002) (Figure 1B). However, more mosquitoes per surface area (i.e., density of mosquitoes caught in 100 cm^2^ of the target) were recovered on small targets (median = 1.0) when compared to large targets (median = 0.3; Mann-Whitney U test, n = 24, U = 454.5, p < 0.001). In the competitive assay where both small and large targets were presented together, mosquitoes exhibited a strong preference for landing on the large target, with an average of 80.0% ± 4.9% SEM of the landings occurring on the large target (median = 5.0), while only 20.0% ± 4.9% SEM of the landings were recorded on the small target (median = 1.5; Wilcoxon test, n = 12, W = 0, p = 0.002) (Figure 1C). The number of visits recorded around targets depended on whether the accompanying target was a different size (generalized linear models (GLM), χ^2^ = 10.98, d.f. = 2, p = 0.004). Mosquitoes visited small targets in the competitive assay significantly fewer times compared to the large target (Tukey’s test, with the large target in the large vs. large assay: t = −2.74, p = 0.03; with the large target in the competitive assay: t = −2.62, p = 0.04; with the small target in the small vs. small assay: t = 3.42, p = 0.003). Thus, when presented together, large targets drastically outcompeted small targets in attracting mosquitoes. However, no difference was found in the number of visits when targets were presented alongside targets of the same size (Tukey’s test, t = 0.81, p = 0.85), indicating that when there is not direct competition, small targets remain highly utilized. Mosquitoes also flew for longer and covered greater distances around small targets when these were the only targets available compared to when small targets were presented alongside large targets (Tukey’s test, for total time: t = 2.97, p = 0.02; for total distance: t = 2.77, p = 0.04) (Figures 1E–1G). Tortuosity, an index that describes whether a flight track is straight (non-tortuous) or whether it has many turns and twists, varied significantly depending on the target type (ANOVA, F_(2,103)_ = 13.65, p < 0.001). Above and in proximity of small targets flight tracks were more tortuous than those recorded around large targets and those around the area where no visual or thermal cue was offered, which we define as a “no-target area” (Tukey’s test, in competitive assay: t = −3.74, p = 0.002; in large vs. large assay: t = −5.27, p < 0.001, with no-target area: t = −4.56, p < 0.001) (Figure 1H). Mosquitoes flew consistently at a slower speed around the targets compared to their speed when flying above the no-target area (ANOVA, F_(2,103)_ = 30.41, p < 0.001) (Table S1) (Figure 1I).

### Target orientation does not affect mosquito landing rate, but in vertical targets downwind surfaces receive more visits of longer distance and duration than upwind surfaces

**Figure 2:**
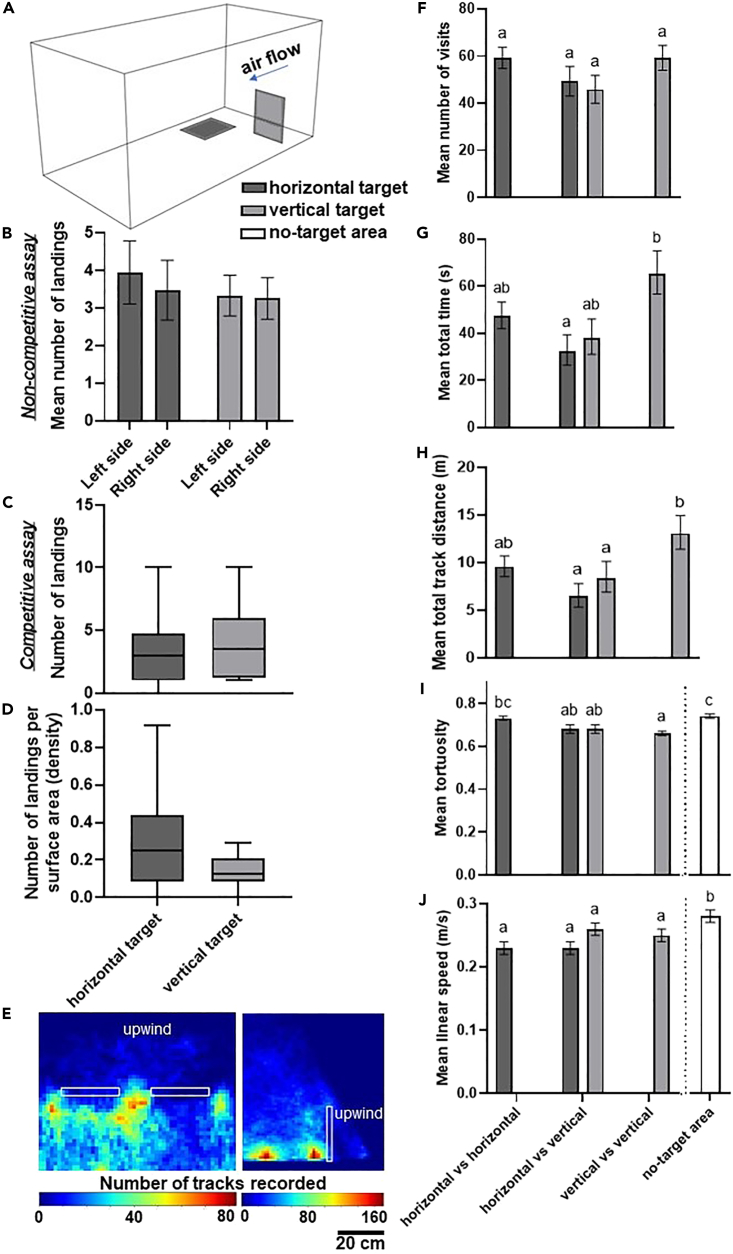
Close-range approach and landing responses of *An.coluzzii* females when presented with targets with same or different spatial orientations (A) Schematic of the wind tunnel with the horizontal (left) and vertical (right) targets. (B) Mean ± SEM number of mosquitoes that landed on the two target types in non-competitive assays. Bars display raw means while statistical analysis (LMM) was performed on log-transformed data. (C) Box-and-whisker plots show the number of mosquitoes that landed on targets in the competitive assay, data analyzed by Wilcoxon signed-rank test. (D) Box-and-whisker plots show the number of mosquitoes that landed per surface area (i.e., density), data pooled from all assays and analyzed by Wilcoxon signed-rank test. (E) Heatmaps of the XY and YZ wind tunnel planes showing the density of tracks recorded around the vertical targets in the vertical vs. vertical assay. White rectangles indicate target position. Note that the vertical targets were visited substantially more on the downwind section compared to the upwind section, and the targets were mostly approached closer to the floor. (F–J) Mean ± SEM of flight track parameters. Data in (F) were analyzed by a GLM followed by Tukey’s post hoc test, while data in (G–J) were analyzed by a two-way ANOVA. For all panels, where present, different lowercase letters indicate significant differences between treatments (p < 0.05). Where the letters are not displayed, no significant difference was found in the statistical analysis.

Mosquito preference for two different spatial orientations (i.e., with the same sized target positioned either vertically so that it stood upright in the column of moving air, or laid flat on the floor, broadly reflecting either a standing or laying host) was tested with three different assays (Figure 2A). Neither the spatial orientation of the target itself nor the spatial orientation of the other target presented alongside had any significant effect on the landing response (LMM, ANOVA, F_(1,70)_ = 0.70, p = 0.41; F_(1,70)_ = 0.13, p = 0.72). No significant difference was found in the number of mosquitoes that landed on either target in non-competitive assays (LMM, ANOVA, F_(1,73)_ = 0.71, p = 0.40), or in competitive assays (Wilcoxon test, n = 12, W = 47, p = 0.55), where 57.3% ± 7.9% SEM mosquitoes had landed on the vertical target (median = 3.5) and 42.7% ± 7.9% SEM on the horizontal target (median = 3.0) (Figures 2B and 2C). Although the number of mosquitoes recovered on both target types was similar, the vertical target caught a significantly lower density of mosquitoes per 100 cm^2^ (vertical target: median = 0.1, n = 24; horizontal target: median = 0.3, n = 34; Mann-Whitney U test, U = 263, p = 0.02); this is likely to be because the vertical target projected into the three-dimensional space of the flight arena and thus provided twice the potential landing area as the horizontal target, which laid flat on the floor. Overall, mosquito flight behavior remained consistent irrespective of target orientation. Independently of the assay and the target type, an equal number of visits were recorded around the different targets throughout the entire experiment (GLM, for assay: χ^2^ = 4.08, d.f. = 2, p = 0.13; for treatment: χ^2^ = 0.11, d.f. = 1, p = 0.74) (Figure 2E). Mosquitoes flew consistently at a significantly lower speed near all targets compared to the no-target area (ANOVA, F_(2,118)_ = 35.97, p < 0.001) (Table S1) (Figure 2I). The total flight duration and the total distance covered depended only on whether the target types were competing or not (ANOVA, F_(2,78)_ = 3.87, p = 0.02; F_(2,78)_ = 3.34, p = 0.04). Specifically, both parameters only differed significantly between the vertical targets in the vertical vs. vertical assay when compared against the horizontal target in the competitive assay (Tukey’s test, for total time: t = 2.89, p = 0.02; for total distance: t = 3.00, p = 0.02). Altogether, the results presented indicate that mosquito flight behavior near a target is not majorly influenced by its orientation. The only parameter that presented a clear difference between targets was tortuosity, as tracks recorded around vertical targets were significantly more tortuous compared to tracks recorded around horizontal targets in non-competitive assays (Tukey’s test, t = −3.89, p = 0.002). Furthermore, tracks recorded around vertical targets and in both vertical and horizontal targets of the competitive assay were significantly more tortuous than tracks recorded in the no-target area (Tukey’s test, t = −6.28, p < 0.001; t = 4.17, p < 0.001; and t = −3.95, p = 0.001, respectively) (Figure 2H). This could be due to the fact that vertical targets created a physical barrier that forced mosquitoes to drastically change their flight trajectory, thus increasing the tortuosity. Focusing on vertical targets only, significantly fewer mosquitoes landed on the upwind surface (median = 1.0) compared to the downwind surface, which caught twice as many mosquitoes (median = 2.0, Wilcoxon test, n = 36, W = 104, p = 0.01). Track analysis showed that the number of visits was significantly greater around the downwind surface (GLM, χ^2^ = 47.22, d.f. = 1, p < 0.001) and that mosquitoes spent more time (ANOVA, F_(1,68)_ = 67.21, p < 0.001) and covered longer distances (ANOVA, F_(1,68)_ = 92.47, p < 0.001) around the downwind surface (Figure S1).

### Modeling distance detection of high contrast visual targets

**Figure 3:**
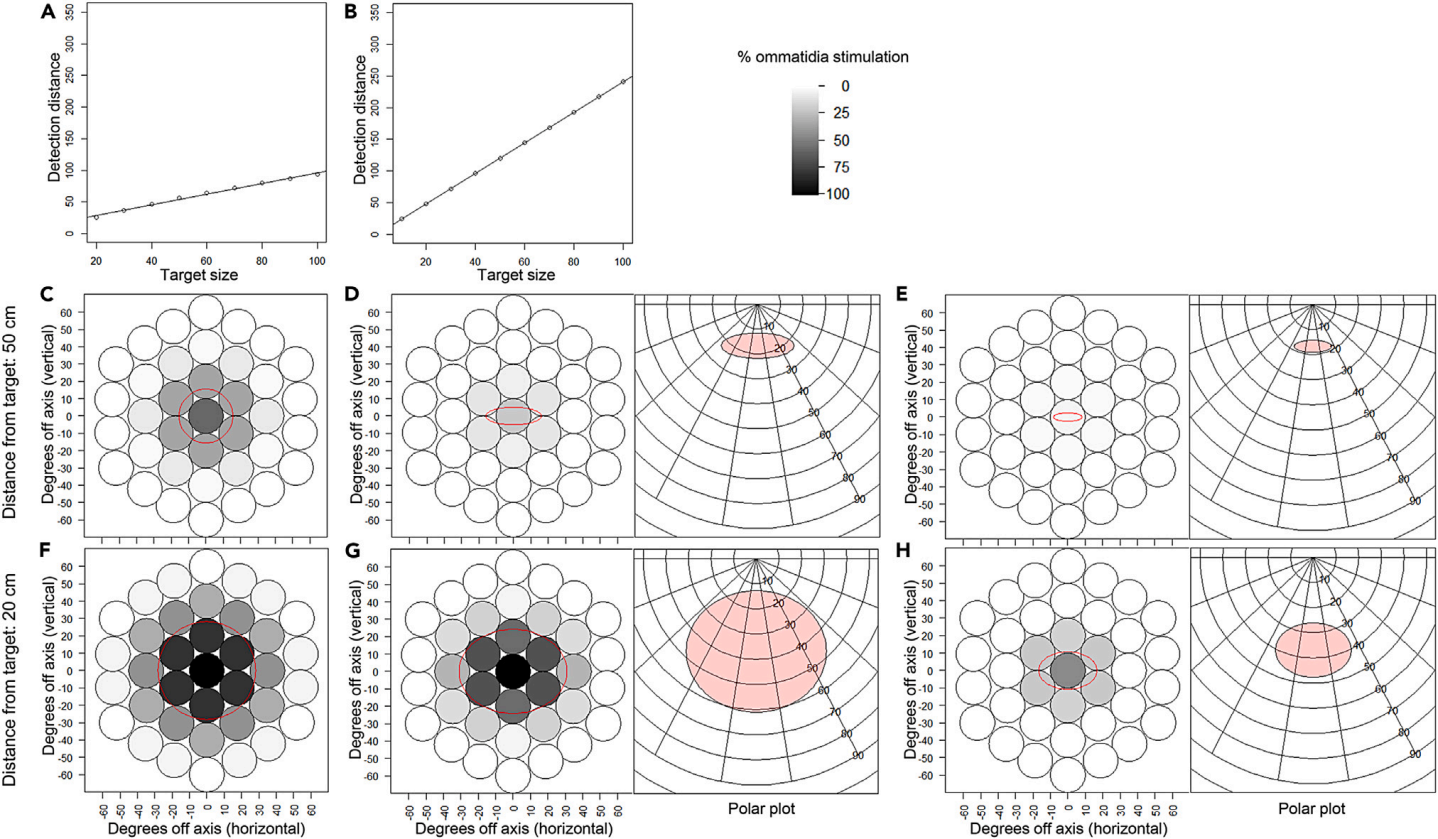
Output of the eye model for visual target resolution in *An.coluzzii* mosquitoes (A) Maximum detection distance for targets of different size positioned horizontally, with mosquitoes flying at 15 cm height above the target. (B) Maximum detection distance for targets of different size positioned vertically. (C–H) Eye model plots showing the level of stimulation of 37 adjacent ommatidia (circles), in response to visual targets (mapped over the ommatidia as red circles). Polar plots show the optical distortion of horizontal targets. (C) A large vertical target (30 cm diameter) positioned 50 cm away. (D) A large horizontal target (30 cm diameter) positioned 50 cm away. (E) A small horizontal target (15 cm diameter) positioned 50 cm away. (F) A large vertical target (30 cm diameter) positioned 20 cm away. (G) A large horizontal target (30 cm diameter) positioned 20 cm away. (H) A small horizontal target (15 cm diameter) positioned 20 cm away. Note that for targets of equal size (C, D) and (F, G), the level of ommatidia stimulation is greater when the target is positioned vertically (C, F) than horizontally (D, G). Model parameters were set as follow: inter-ommatidial angle: 8°, receptive field: 40°, altitude (for horizontal targets only): 15 cm, individual ommatidia threshold: 20, total eye threshold: 100%.

To estimate the maximum distance at which mosquitoes could detect visual targets, we developed a software-based model that calculates the proportion of ommatidia that are stimulated by circular targets. The ommatidia (singular: ommatidium) are the repeated photoreceptor units that make up the compound eye, each eye having about 200–300 ommatidia. Each ommatidium is composed of a corneal lens, a crystalline cone, and eight photoreceptor cells.^39^ The model accounts for targets of different sizes, placed at different distances, and in different spatial orientations, and bases its parameters on published optical properties of mosquito ommatidia.^40^ Assuming an interommatidial angle of 8°, a receptive field of 40°, and a flight height of 15 cm above the ground, the model indicates that the large horizontal target is resolved by *An. coluzzii* eyes ∼39 cm away, and the small horizontal target at ∼ 25 cm. When repeated for vertical targets, the large target is resolved ∼72 cm away, which is nearly double the distance at which the same target would be resolved if horizontal; while a vertical small target is resolved at a distance of ∼36 cm, which is 30% further away than if the target was horizontal. This is because targets stimulate more ommatidia when they are placed vertically compared to when they are placed horizontally (Figures 3C, 3D, 3F, and 3G). According to our model, mosquito’s flight height can also subtly influence the distance of resolution; a large horizontal target would be resolved ∼39 cm away to mosquitoes flying at a height of 25 cm, and ∼37 cm away if at a flight height of 15 cm. Taken together, we conclude that target size is directly proportional to the distance at which it is resolved, with larger targets resolved at a considerably greater distance than smaller targets, while targets with comparable sizes are detected further away if oriented vertically rather than horizontally.

### Mosquito landing rate is directly proportional to area of thermal stimulus

**Figure 4:**
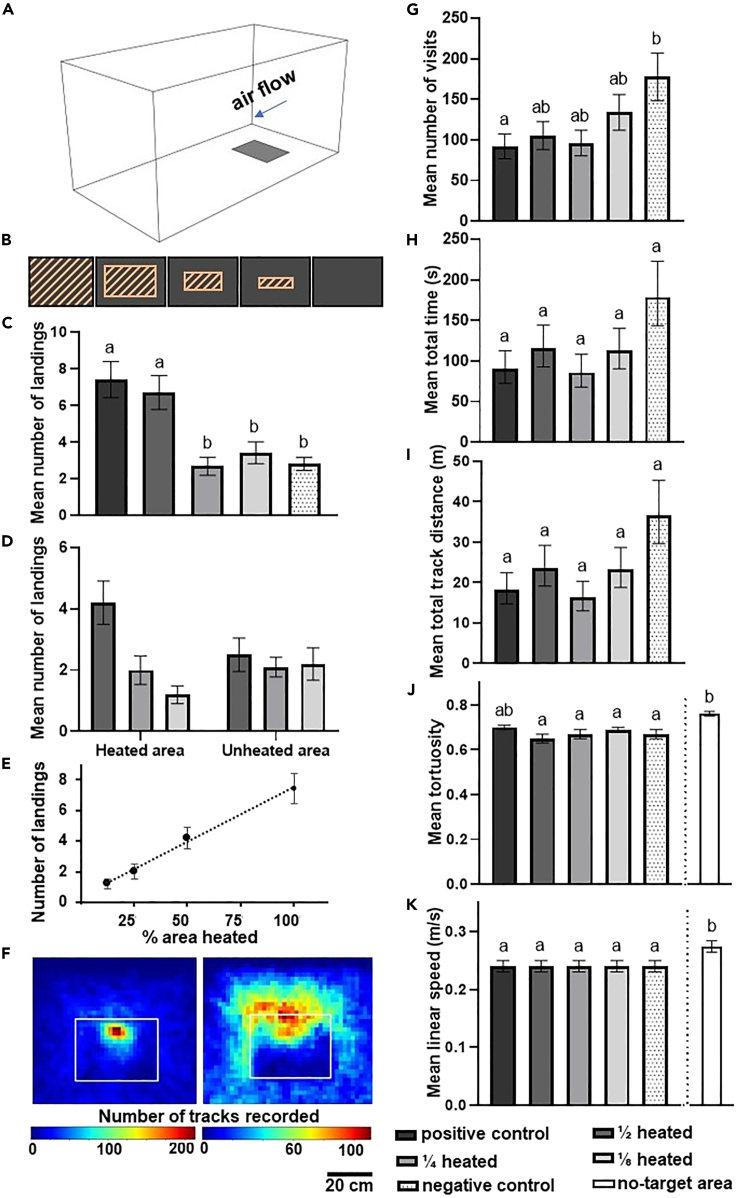
Close-range approach and landing responses of *An.coluzzii* females when presented with different thermal signature targets (A) Schematic of the wind tunnel with the target. (B) Schematic representation of the different target types with the different thermal signatures; red diagonal stripes represent the heated area in each of the five treatments. (C) Mean ± SEM number of mosquitoes that landed on the five target types, data analyzed by GLM followed by Tukey’s post hoc test. (D) Mean ± SEM number of mosquitoes recovered from heated and unheated target areas. (E) Mean ± SEM number of mosquitoes recovered on the heated area for all target types. (F) Heatmaps of the XY wind tunnel plane showing the density of tracks recorded around the positive (left) and negative (right) control targets. White rectangles indicate target position. (G–K) Mean ± SEM of flight track parameters. Data in (G) were analyzed by a GLM followed by Tukey’s post hoc test, while data in (H–K) were analyzed by a one-way ANOVA. For all panels, where present, different lowercase letters indicate significant differences between treatments (p < 0.05).

To understand how landing behavior may be affected by the spatial extent of a thermal stimulus, we manipulated the area of a target which emitted heat. Based on the results described previously, mosquitoes were presented with a large horizontal target with high visual contrast, where the surface was either fully heated (positive control), half heated, one-quarter heated, one-eighth heated, or not heated at all (negative control). The different treatments had a significant effect on *An. coluzzii* landing behavior (GLM, χ^2^ = 42.79, d.f. = 4, p < 0.001) (Figure 4C). Results cluster in two distinct groups: the positive control and the half-heated target elicited a similar landing rate, while the rest of the treatments elicited a significantly lower landing rate. A clear linear relationship was found between the mean number of mosquitoes that landed on the heated areas of the targets and the size of the thermal signature, indicating landing is directly proportional to the area of the thermal signature (Figure 4E). Statistical comparisons of landing rate were standardized by density (i.e., number of mosquitoes landing per cm^2^ of target surface), as different sized areas were heated and unheated. No significant difference was found when comparing the densities of mosquitoes that landed on the central heated area with the unheated margins for the half-heated target (median for central area = 0.7, median for margin area = 0.4; Wilcoxon test, n = 10, W = 36, p = 0.12) and the one-quarter heated target (median for central area = 0.3, median for margin area = 0.1; Wilcoxon test, n = 9, W = 37.5, p = 0.09). In contrast, significantly more mosquitoes landed in the heated area (median = 1.0) than the unheated area (median = 0.1) of the one-eighth heated target (Wilcoxon test, n = 10, W = 48, p = 0.04), despite the latter having a considerably larger area. Furthermore, in the one-quarter heated, one-eighth heated, and the negative control, the mean density of mosquitoes recovered on the unheated margins was comparable in all three treatments (∼ mean ± SEM = 0.2 ± 0.05) although these areas were of considerably different sizes (Table S2). Overall, the number of mosquitoes landing on the heated area strongly depended on the extent of its area, while the number of mosquitoes landing on the unheated area did not differ between treatments (LMM, interaction term between treatment and part of the target, ANOVA, F_(2,44)_ = 4.40, p = 0.02) (Figure 4D). Thus, it appears that the size of the unheated area does not affect the number of landing mosquitoes while the size of the heated area plays an important role in increasing the number of landings. By using the mean density recovered from the whole target surface as a proxy to estimate target efficiency, it arises that the positive control and the half-heated target are on average approximately twice as efficient at trapping mosquitoes compared to the targets where only one-quarter and one-eighth of the surface was heated, while also being on average approximately 2.5 times more efficient compared to the negative control (Table S2). The flight behaviors of mosquitoes around all targets in this experiment were remarkably consistent. The mean speed of tracks recorded around the target was similar across all treatments and was significantly lower compared to the linear speed of the tracks recorded around the no-target area (one-way ANOVA, F = 7.09, d.f. = 5, 92, p < 0.001) (Figure 4K) (Table S1). No statistical difference was found in the total flight duration (ANOVA, F _(4,44)_ = 1.70, p = 0.17) and distance (ANOVA, F_(4,44)_ = 2.13, p = 0.09) of tracks recorded around different treatments. Similarly, no difference was detected in the tortuosity of track recorded around different treatments, and a significant difference was found only when results of the no-target area were introduced in the model (ANOVA, F_(5,92)_ = 9.72, p < 0.001) (Figures 4H–4J). This suggests that these parameters are minimally influenced by variations in the thermal signature’s area. The negative control target received significantly more visits compared to the positive control (Tukey’s test, t = −2.81, p = 0.04), which may be because mosquitoes returned multiple times without being induced to land in the absence of heat (Figure 4G).

## Discussion

In this study, we have shown that the physical characteristics of a target significantly influence the landing responses and some flight characteristics of host-seeking *An. coluzzii*. These findings are directly relevant to efforts to design features of devices for mosquito control and surveillance. Such features could both maximize mosquito attraction and prompt the sequence of behaviors that triggers close-proximity approaches or landing, with the effect of bringing vectors within range of the devices’ killing or capturing mechanisms. This lays the foundation for systematic testing of and improvements in target characteristics of malaria vector-trapping devices.

With a consistent odor stimulus in the background, mosquitoes land more on large targets and visit them more when in competition with small targets. Landing behaviors are further modulated by not only the presence or absence of a thermal cue^13^^,^^20^ but also increase as the heated area increases. Below a certain area, a thermal cue set at host temperature seems to be insufficient to trigger landing as when this covers less than half the target’s area, relatively few mosquitoes land. In contrast, *Ae. aegypti* females land on small Peltier plates (6 cm × 9 cm) when exposed to carbon dioxide^23^ or on small (∼5.5 cm–7.5 cm diameter) landing areas that presented different combinations of odor, heat, and visual cues.^22^ This difference may be due to the area size in which the landing target was presented, as in the present study mosquitoes were allowed to freely fly in a large wind tunnel, thus reducing the probability of finding the target by pure chance, or it might be due to differences in the innate behaviors of the two species.

In terms of potential applications, a number of new malaria vector surveillance and control tools could be improved by design modifications that incorporate quantified behavioral responses of *Anopheles* to increase capture/kill efficiency or make them more practical for use at large scale without loss of efficacy.^41^ The fact that mosquitoes landed in similar numbers on fully heated and half-heated targets provides the basis for trap optimization. Thermal cues augment trap catches;^13^ however, they can prove difficult or expensive to implement in the field.^8^ Thus, being able to halve the area emitting heat could drastically reduce associated production and running costs without any significant reduction in capture rates. Furthermore, considering that unheated margins of partially heated targets received similar landing rates, irrespective of their area, the overall size of the visual target if not accompanied with adequate thermal cues may not be a dominant factor that defines landing. Therefore, to create effective targets, it might be important to consider not only their final size but also specifically the surface areas covered by each physical cue.

We found that *An. coluzzii* exhibit a landing preference for large targets over small targets. However, strong differences in attraction and flight behavior mostly occur when different size targets are in direct competition. This may be explained by the distance at which each target is detectable by the insect’s visual system. Assuming a dark-adapted eye, which has a resolution angle of approximately 40° in *Anopheles*,^40^ the estimated distance at which mosquitoes could resolve large horizontal targets is approx. 1.5 times the distance at which small targets would be resolvable. With an average flight speed of ∼0.24 m s^−1^, mosquitoes flying upwind toward the odor source would therefore resolve the outline of the larger horizontal target about 0.5 s before the smaller horizontal target, and 1.5 s before if both targets are vertical. Thus, larger targets could attract mosquitoes from a wider area by initiating attraction from a greater distance. However, over close-range distances at least, the presence of competitor sources of attraction could diminish the effectiveness of lure-and-kill targets, as has been suggested as a possible explanation for poor field performance of traps.^42^ Our results suggest size may be an important parameter in driving competition between targets. Thus, careful positioning of mass trapping and surveillance devices will be needed to limit the influence of competing attractants, both olfactory and visual. This will be particularly relevant to the development of “push/pull” strategies, where attractive “pull” components, such as baited traps, are relatively weak at luring mosquitoes toward them^12^ and appear to be affected by competing stimuli.^42^ Our results indicate “pull” devices have the potential to be improved with the addition of visually conspicuous features and heat; optimizing them and studying the effects of competition between traps and against real hosts could be a fruitful area for further research.

Although small targets induce fewer total landings, they still elicit high levels of overall attraction toward the vicinity of the target and mosquitoes repeatedly turn above them. This suggests intensive exploratory behavior which is associated with assessment of the stimulus,^43^ but is a surprise given the generally poor resolution of anopheline eyes,^40^ and provides further evidence that despite their nocturnal environment the visual system of *An. coluzzii* is well adapted to make use of even a small (<20 cm wide) visual stimulus to locate potential hosts.^39^ Large targets with small heated areas did not stimulate many landings, but retained a high level of attraction characterized by repeated visits of long duration, indicating that while large thermal and visual cues eliciting greater overall attraction and landing, smaller areas of visual and thermal stimuli are still sufficiently attractive to lure mosquitoes into close proximity of a target. This has important implications for the design of control tools; those that require direct physical contact with, for instance, an insecticide, should incorporate large thermal and visual cues to maximize landing, while for traps that only require mosquitoes to fly within a certain distance of a counter-flow current or other capture/kill mechanism,^8^ small, strategically placed visual and thermal cues could suffice.

Mosquitoes are strongly attracted to convection currents that originate from warm bodies,^31^^,^^44^^,^^45^ and the area occupied by these currents may vary depending on the body’s orientation; a horizontal target would produce a larger column of rising warm air compared to that from the slim footprint of a flat vertical target. *Anopheles* spp. females were reported to mostly approach and land on the top (horizontal) section of human-baited bed nets,^46^ whereas we found neither a preference for landing on either vertical or horizontal targets, nor any differences in the number of visits, the total track duration, nor the total distance flown. This may be because we used human odors introduced in a laminar airflow upwind of the targets rather than emanating from the targets themselves, thus limiting multimodal host location behavior.^47^ The only difference we observed was greater tortuosity of flights around vertical targets, which is unsurprising as these create a physical barrier that forces mosquitoes to drastically change their trajectory to avoid collision. Results from diurnal mosquitoes show a preference, but this differs by species. *Aedes albopictus* displayed lower landing rates on a feeding substrate kept at 37°C if this was positioned vertically rather than horizontally on the floor^48^; conversely, *Ae. aegypti* land nearly ten times more on a vertical, purple unheated target compared to the same target positioned horizontally.^49^ The remarkable discrepancies in the results reported on this topic highlight the need to further investigate the effect of the spatial orientation on landing behavior across the Culicidae.

The computational model of the mosquito visual system presented here provides the means to estimate the proportion of ommatidia stimulated by targets of different sizes and placed at different distances and in different spatial orientations, while also estimating the maximum distance at which different targets could be detected by mosquitoes. This offers practitioners an estimate of the maximal distance at which a visual target might attract mosquitoes. We encourage practitioners to use the model by adjusting the parameters according to (1) the anatomical and physiological traits of different nocturnal or crepuscular mosquito species and (2) the characteristics of different target configurations. However, these inferences are based on assumptions derived from the literature on mosquito optics and as such provide only a theoretical indication of visual detection distance. Future work to determine these experimentally could be used to robustly test model predictions.

Taken together, results from the three experiments show that the proportion of landing mosquitoes did not increase in a manner that corresponded with the increment of the surface exposed. In the first experiment, large targets caught only double the number of mosquitoes compared to small targets, despite offering four times the trapping area, corresponding to results for *G. palpalis* and *G. fuscipes*.^36^ In the spatial orientation experiment, the vertical target caught a similar number of mosquitoes compared to the horizontal surface, although it presented double the area exposed. Furthermore, the number of mosquitoes landing on two large targets presented simultaneously in experiment one was similar to the number recovered on the single fully heated target in experiment three. This implies that independently of the number of targets presented and of the area of the trapping surface exposed, a similar proportion of mosquitoes responded to the cues and landed on the target/targets, and a similar number of mosquitoes remained unresponsive. In practical terms, this indicates that doubling the number of targets or doubling target size one will not necessarily catch double the number of mosquitoes and, as with tsetse flies,^36^ larger targets may not necessarily be the most efficient. To intensify control efforts, future research that includes an in-depth cost-effectiveness study similar to that carried out for tsetse traps^35^ alongside systematic trap optimization^34^ is recommended.

Altogether, this study reveals the relative effects that variations in individual physical stimuli have on mosquito close-range host-seeking behavior, and how these play specific roles in attracting and subsequently eliciting landing in *An. coluzzii*. Our results not only further the understanding of this neglected topic, but also identifies that the area of visual and thermal stimuli are factors to incorporate into optimized designs of traps and targets for testing as vector surveillance and control tools. Here, we show that minor variations in these characteristics can cause significantly different behavioral responses, which in turn could influence the efficiency of vector control tools.

### Limitations of the study

This research was conducted under controlled laboratory conditions, using laboratory strains of mosquitoes. These methods offered control over experimental conditions but constrained mosquitoes to an artificial environment within a limited physical area. In the wild, mosquitoes navigate an extensive and dynamic environment containing multiple, complex, overlaid sources of stimuli. Thus, further semi-field or field-based research should be carried out to corroborate the findings reported here and help to fine-tune our understanding of mosquito behavioral responses to potential targets in real-world scenarios, which will be of utmost importance in developing vector control tools. As different anopheline mosquitoes may exhibit different behavioral traits, we also recommend performing these experiments using different species of malaria vectors.

## STAR★Methods

### Key resources table

**Table:** 

REAGENT or RESOURCE	SOURCE	IDENTIFIER
**Chemicals, peptides, and recombinant proteins**		

Aquarium salts	Tropic Marin	Pro-Reef
Organic baby rice	Aptamil	4-6+ months
Fish food	TetraMin	Tropical flakes

**Experimental models: Organisms/strains**		

*Anopheles coluzzii*	Institut de Recherche en Sciences de la Santé	N/A

**Software and algorithms**		

R software (version 4.1.2)	R Foundation	https://www.r-project.org/foundation/
GraphPad Prism (version 9.0.0)	GraphPad software Inc	https://www.graphpad.com/
Track Analysis	This paper	https://doi.org/10.5281/zenodo.10092390
Mosquito Eye model	This paper	https://doi.org/10.5281/zenodo.10092123
TrackIt3D (version 3.0)	SciTrackS GmbH	N/A

**Other**		

IR LEDs	JC	N/A
Analogue cameras	Basler	acA2440 – 75 μm
Lenses	Fujinon	HF6XA 5M
IR filters for camera lenses	Midopt	LP830 band pass
IR transmitting black acrylic sheets for targets and optomotor squares	Southern Acrylics	N/A
Adhesive film	Barrettine	FICSFILM

### Resource availability

#### Lead contact

All requests for additional information and resources should be directed to Manuela Carnaghi (manuela.carnaghi@greenwich.ac.uk).

#### Materials availability

This study did not generate new unique reagents.

#### Data and code availability

• Data reported in this paper will be shared by the lead contact upon request.
• All original code has been deposited at Zenodo and is publicly available as of the date of publication. DOIs are listed in the key resources table.
• Any additional information required to reanalyze the data reported in this paper is available from the lead contact upon request.

### Experimental model and study participant details

#### Animals

This study used *An. coluzzii* mosquitoes. The colony was established at NRI’s laboratory in 2017 from eggs donated by the Institut de Recherche en Sciences de la Santé, Burkina Faso. Mosquitoes were identified to species level by PCR. The colony was kept in a climate-controlled laboratory set at 26 ± 2°C, 60 ± 10% RH, and 12:12h LD cycle. Adult females were fed on human arms for two non-consecutive days, and eggs were collected in oviposition cups. Eggs were transferred to larval trays that contained 1 L of deionised water solution with 0.1% aquarium salts (Tropic Marin, Germany) and were fed powdered organic baby rice (4–6+ months, Aptamil©, Netherlands) and fish food (TetraMin©, Tetra Werke, Germany) until pupation. Pupae were collected daily and were placed in pupae dishes, which were then enclosed in cages where the adults were kept (approx. 200 adults per cage). Each assay used 25 non-blood feed females, five to ten days old, which prior the assay were starved for 4 h and were kept in darkness for 1 h to allow eye adaptation to low light levels. All assays were conducted in the second and third hour of the mosquitoes scotophase, i.e., the second and third hour of the dark phase in the mosquito light cycle.

### Method details

#### Wind tunnel

The experiments were performed in a large flight arena (1.2 m tall × 1.2 m wide × 2 m long) which permitted mosquitoes to execute flight maneuvers (Figure S2). The arena floor and walls were constructed with white opaque Perspex, whilst the roof was made of a sheet of transparent Perspex, to permit the cameras to detect mosquitoes’ silhouettes inside the arena. The arena was kept at 25 ± 2°C and 65 ± 5% RH. The air pushed into the flight arena was drawn from outside the building by an impelling fan positioned at the upwind end of the wind tunnel; the air was purified by activated charcoal filters, humidified to 65% RH, heated to 25°C, and pushed through a cotton screen, which ensured a laminar airflow. At the downwind end of the wind tunnel an extractor fan pulled air out of the laboratory. This created a constant flow of air of approx. 0.2 ms^−1^ from the upwind end to the downwind end. During experiments the laboratory lights were turned off and the only source of illumination consisted of an array of warm white LEDs placed on the floor, underneath the floor of the flight arena. This provided a homogeneous light level of 0.001 Wm^-2^, which is comparable to the light level mosquitoes experience in the field during full moon nights, at between 420 and 680 nm. To allow optomotor navigation, nine small squares (10 cm × 10 cm) and two large squares (20 cm × 20 cm) of black-coloured infrared (IR) transmitting plastic tiles (Southern Acrylics, UK) were placed randomly on the floor of the wind tunnel. Mosquitoes were released at the downwind end of the wind tunnel from a release cage (15 cm × 15 cm × 15 cm) which was centered with the odor stimulus releasing point.

#### Tracking system

Twenty-eight arrays of twelve high-power IR LEDs with 90° beam angle (JC, UK) were arranged to create a diffuse and evenly distributed IR background around the wind tunnel. Mosquito 3D flight tracks were obtained using TrackIt3D 3.0^50^ and two high-resolution analogue cameras (acA2440 – 75 μm, Basler, Germany) operating at 50 fps and fitted with HF6XA-5M lenses (Fujinon, Japan) and IR filters (LP830 band pass, Midopt, USA), thereby detecting changes in illumination >800 nm. The cameras were suspended ∼60 cm above the flight arena, with a field of view equivalent to the entire flight arena. The IR transmitting plastic tiles described above were visible to mosquitoes but allowed IR light to pass through for detection by the cameras, allowing mosquitoes to be tracked when flying over them. Data were processed using TrackIt3D post-processing software and a custom-made Python program that filtered out erroneous data points, interpolated up to five consecutive missing coordinates, and smoothed the tracks using an ad hoc spline function based on mosquito flight parameters.^51^ Only tracks that entered cuboids encompassing areas where targets were positioned plus a 10 cm buffer in the X- and Y axes were included in analysis. The height of the cuboids was 30 cm for horizontal targets and 60 cm for vertical targets. The following track parameters were analyzed: total number of visits, total time spent in the cuboid, total 3D distance covered in the cuboid, track tortuosity, and track speed (Table S3). To determine the effect of targets on certain flight parameters, a negative control (referred to as “no-target area”) consisting of a cuboid with the same dimensions as the large horizontal target was created in the downwind end of the wind tunnel, away from the target stimulus.

#### Landing target

When not otherwise specified, the landing targets were placed horizontally on the wind tunnel floor and were made of 3 mm thick IR transmitting black acrylic sheets (Southern Acrylics, UK) over which rested transparent plastic bags filled with water that provided the thermal cue. The plastic bags were custom made according to the size and shape of the target and were filled with an adjusted volume of water so that the final thickness of the target was 3 cm. Where needed, the plastic bags were heated in a water bath until they reached a temperature of 38 ± 2.00°C, which allowed for the bags to cool slightly during the experiment and still emit a thermal cue similar to human body temperature and be behaviorally stimulatory. To capture landing mosquitoes, targets were covered with one layer of transparent adhesive film (FICSFILM, Barrettine, UK).

##### For Experiment 1 (target size)

Two rectangular targets were used; a large target of 30 cm × 40 cm, total area = 1200 cm^2^ and a small target of 15 cm × 20 cm, total area = 300 cm^2^. The full surface area of both targets was heated.

##### For Experiment 2 (target orientation)

Two large targets were used throughout, placed either lying flat on the wind tunnel floor with the long sides parallel to the air flow, or vertically at a 90° angle to the wind tunnel floor, with a short side in contact with the floor and held upright in a transparent stand, with the plane perpendicular to the air flow. The entire surface of both targets was heated; the vertical target was therefore heated on both faces of the target.

##### For Experiment 3 (target heat signature)

One large target placed on the wind tunnel floor was used throughout. The area of the target surface that was heated changed to provide one of five treatments: fully heated, where all the surface area was heated (positive control); half of the area heated; one-quarter heated; one-eighth heated; and no area heated (negative control). From this, heated and unheated areas were calculated (see below). The aerial layout of heated and unheated areas is shown in Figure 4B, where unheated areas were maintained using water bags at ambient temperatures, whereas heated water bags were positioned in the center of the surface to provide the heated area.

Different treatments used in Experiment 3 and the corresponding sizes of plastic bags used to compose the target

**Table:** 

Treatment	Size of central (heated) surface	Sizes of lateral (room temperature) surfaces
All area heated (positive control)	1200 cm^2^	N/A
½ area heated	600 cm^2^	600 cm^2^
¼ area heated	300 cm^2^	900 cm^2^
⅛ area heated	150 cm^2^	1050 cm^2^
No area heated (negative control)	N/A	1200 cm^2^

#### Experimental procedure

All experiments were conducted in the presence of host odor, i.e., carbon dioxide and human body odor, which was released at the upwind side of the wind tunnel (Figure S2). The carbon dioxide was introduced in a pulsed manner (8 s on and 7 s off), whilst the human body odor was collected on 100% polyamide nylon socks, which were worn by a volunteer for 24 h. To limit variations in body odor, the socks were worn by the same person who abstained from eating food with spices, drinking alcohol, and using perfumes or strong perfumed soaps and clothes detergents. Experimental mosquitoes were transferred into the release cage 10 min before each assay started. With the odor cues initiated, the release cage was opened remotely, and video recording started. The experimenter left the room to avoid odor contamination and only returned after 15 min, when the assay was terminated and the number of mosquitoes recovered in different parts of the wind tunnel and on the targets was recorded. For vertical targets the number of catches on the downwind side (facing the release cage) and on the upwind side (facing toward the incoming air and odor sources) was noted, and in Experiment 3, the number of mosquitoes landing on the heated area and on the unheated margins of the target were also recorded. Surgical gloves were worn throughout the experimental procedure to avoid odor contamination.^43^ At the beginning of each experimental week the wind tunnel surfaces were washed with deionised water, wiped with pure ethanol and were then left to air dry whilst the fabric components were washed at high temperatures (>65°C) with a fragrance-free detergent.

##### Experiment 1 (target size)

Two-choice assays were carried out to determine responses to and preferences for large and small targets: 1) small vs. small (two small targets presented together), 2) large vs. large (two large targets presented together), and 3) large vs. small (a large target presented alongside a small target, hereafter referred to as “competitive assay”). Targets were positioned 30 cm from the upwind net (Y axis), small targets were placed 15 cm from the lateral walls (X axis); large targets were 13 cm from the lateral walls.

##### Experiment 2 (target orientation)

Two-choice assays to determine responses to and preferences for vertical and horizontal targets were carried out: 1) vertical vs. vertical (two vertical targets presented together), 2) horizontal vs. horizontal (two horizontal targets presented together), and 3) vertical vs. horizontal (vertical target presented alongside a horizontal target, hereafter referred to as “competitive assay”). In all assays, targets were positioned 30 cm from the upwind net (Y axis), with 20 cm in between them and both being located 13 cm from the lateral wind tunnel walls.

##### Experiment 3 (target heat signature)

No-choice assays to determine the behavioral effect of the proportion of target area that is heated were carried out. A single large target was placed in the center of the X axis and 30 cm from the upwind net (Y axis) with the long side parallel to the net.

Figure S2

### Quantification and statistical analysis

Twelve replicates of Experiment 1 and ten replicates of Experiment 3 were carried out for each assay type. For Experiment 2, twelve replicates were carried out for the horizontal vs. vertical assay, and the vertical vs. vertical assay, while for the horizontal vs. horizontal assay one replicate was carried out per experimental day, and these data were combined with the 12 replicates obtained in the large vs. large assay in Experiment 1, as the two assays presented the same targets and conditions (17 total replicates). Assays within the same experiment were tested in a quasi-randomised order, between and within days to control for the effect of testing sequence. Rotation of targets between left or right position in the wind tunnel was quasi-randomised to exclude the effect of position bias. A total of 114 assays were conducted using 2855 mosquitoes. Throughout all experiments the average percentage of mosquitoes activated was 68.3 ± 1.4% SEM, the range of activation in each assay was within ±10% of this number.

Statistical analyses were performed using RStudio^52^ and R packages “lme4” for the LMM,^53^ “MASS” for the GLM,^54^ and “multcomp” for Tukey’s multiple comparisons.^55^ For Experiment 1 and Experiment 2, an LMM on log-transformed data were used to analyze differences in the number of landings on different target surfaces throughout all treatments and assays. The number of mosquitoes landing on the target was a dependent factor, whilst the treatment, the type of the other target presented simultaneously, and the side where the target was positioned within the wind tunnel, were independent factors. As the factor side was not significant it was removed from the model. Replicates were entered as a random factor to control for pseudoreplication associated with the experimental design. After confirming compliance with the test assumptions, an ANOVA was used on the LMM and p-values were extracted using an ad hoc function (anomer), where the residual degrees of freedom were adjusted to allow for the design effect, calculated from the intra-cluster correlation coefficient. To compare the number of landings on the upwind surface versus the downwind surface of vertical targets, and landing preference in competitive assays in Experiment 1 and 2, a Wilcoxon signed-rank test for related samples was used.

Data from Experiment 3 were analyzed using a GLM with negative binomial errors and log link. Differences in the number of mosquitoes found on the targets in different treatments were assessed using a one-way analysis of deviance, followed by multiple comparisons of means with Tukey’s tests.

Additional control for pseudoreplication was provided in all three Experiments by the nature of the target, as once a mosquito touched the target (i.e., landed on the target) it remained glued on it. This meant that once a mosquito completed the host-seeking behavior making its final descend on the target it remained stuck, and it was therefore removed from the pool of mosquitoes available in the arena. The tracking system recorded the mosquito as “landed” and it then stopped recording its position, to avoid skewing the data. For the final count of the number of mosquitoes that landed, we counted only the number of mosquitoes that were caught on the trap surface. The influence that each mosquito had on other mosquitoes’ behavior was deemed negligible given the large size of the flight arena and the targets. To standardise the number of landings on different targets independently of the area of the exposed surface, densities were calculated by dividing the raw number of mosquitoes recovered on a target by the area of the target in cm^2^. For Experiment 1 and 2 the differences in densities between different target were analyzed using Mann Whitney U test for independent sample as the compared densities came from separate assays, whilst to analyze the differences in densities between the non-heated margin areas and the heated central areas in Experiment 3 a Wilcoxon signed-rank test for related samples was used.

For all three experiments, mosquito age in days was added to the existing statistical models as a factor. An analysis of deviance found its introduction did not produce any significant change in the model. In all statistical analyses, the criterion for statistical significance was p < 0.05.

#### Analysis of 3D tracking data

Within each experiment, the number of times a target was visited were compared using a GLM with negative binomial errors and log link followed by Tukey’s post hoc test. Target and assay type were introduced into the model as independent factors, as were side (upwind or downwind), where relevant. The factor side (intended as target positioned left or right in the wind tunnel) had no effect on flight parameters and was therefore excluded from the model.

The remaining parameters obtained from the tracking analysis were compared between treatments and assays from the same experiment using a two-way ANOVA for Experiment 1 and 2 (with assay and treatment as independent variables), and a one-way ANOVA for Experiment 3. Data were assessed prior to the analysis to ensure that ANOVA’s assumptions were respected, and for total time and total distance data were log-transformed prior to analysis. For tortuosity and linear speed, data obtained from the treatment cuboid were compared with that from the no-target area.

#### Eye model

The eye model was programmed in R^52^ using packages “conicfit”,^56^ “gpclib”,^57^ and “plotrix”.^58^ The model represents an eye as a group of 37 adjacent ommatidia. For each ommatidium, the program calculates the percentage of stimulation, which is defined as the percentage of the receptive field of each ommatidium that is filled by the target. To calculate the maximum detection distance, the model calculates the distance at which the total eye stimulation, counted as the sum of the individual ommatidium stimulation for all ommatidia whose receptive fields are stimulated over the selected threshold value, reaches the value of 100%. More information on the model structure, assumptions, outcomes, and instructions can be found in the User Manual, which is deposited together with the program script on Zenodo.
