# Supplementary material for: Visual and thermal stimuli modulate mosquito-host contact with implications for improving malaria vector control tools
Source: iScience. 2023 Nov 27;27(1):108578. doi: 10.1016/j.isci.2023.108578 (PMC10753043; doi:10.1016/j.isci.2023.108578)
Supplement: Document S1. Figures S1, S2, and Tables S1–S3 [file mmc1.pdf]

iScience, Volume *27*

Supplemental information

Visual and thermal stimuli modulate mosquito-host contact with implications for improving malaria vector control tools

Manuela Carnaghi, Federico Mandelli, Lionel Feugère, Jillian Joiner, Stephen Young, Steven R. Belmain, Richard J. Hopkins, and Frances M. Hawkes

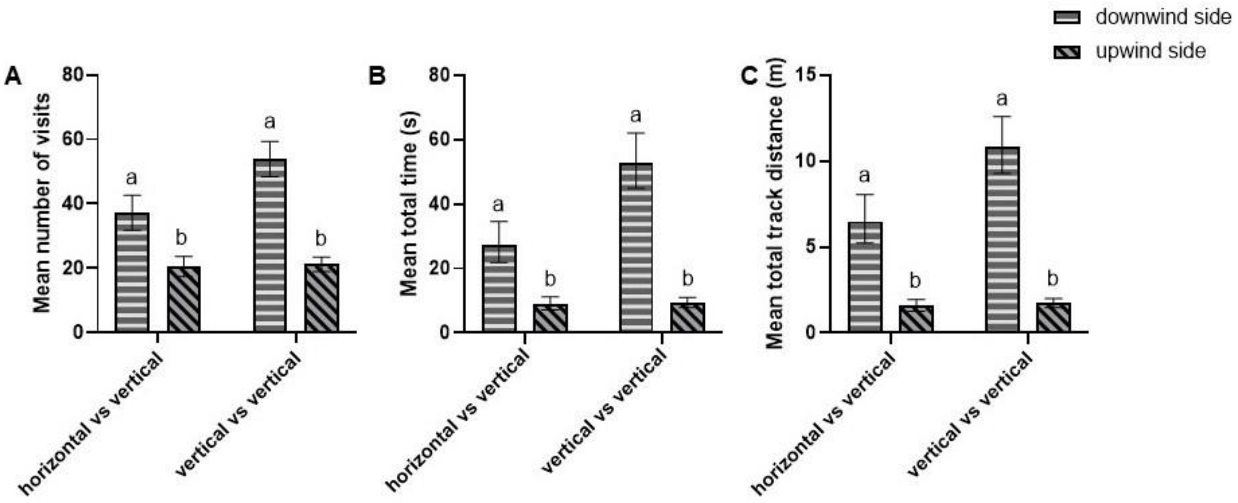

Figure S1. Comparison of track parameters recorded downwind and upwind around vertical targets in Experiment 2, related to Figure 2. (A) Mean ± SEM of number of visits, data analysed by GLM followed by Tukey's post hoc test. (B) Mean ± SEM of the total flight track time analysed by a two-way ANOVA. (C) Mean ± SEM of the total track distance, analysed by a two-way ANOVA. For all panels different lowercase letters indicate significant differences between treatments ($P<0.05$).

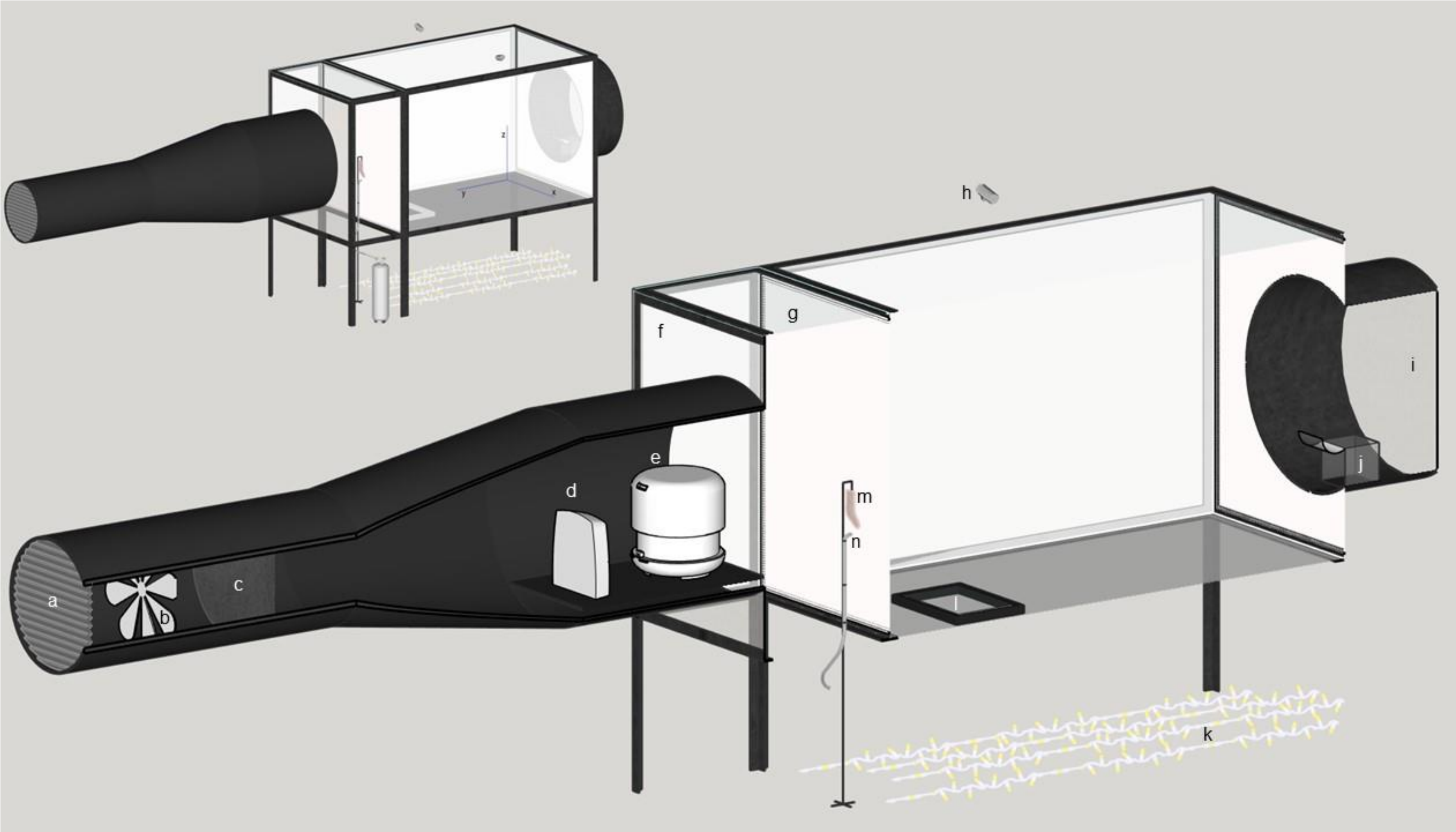

Figure S2. Wind tunnel and flight arena schematic, related to STAR Methods. Experimental area view on its entirety and as a cross section, where the different components can be seen: (a) shutter, (b) fan, (c) filter, (d) heater, (e) atomising humidifier, (f) brushed cotton screen, (g) upwind white net screen, (h) camera connected to 3D tracking system, (i) downwind white net screen, (j) release cage, (k) lighting array, (l) landing target, host odour delivery platform with (m) the worn sock and (n) the carbon dioxide delivery tube.

Table S1. Turkey's test comparison of the linear flight speeds recorded around different targets throughout the three experiments, related to Figure 1, 2, and 4.

Experiment	Target of comparison 1	Target of comparison 2	t-value	P-value
1	Large target in the large vs large assay	Neutral box	6.14	<0.001
1	Large target in the competitive assay	Neutral box	3.54	0.005
1	Small target in the competitive assay	Neutral box	-4.44	<0.001
1	Small target in the small vs small assay	Neutral box	-6.80	<0.001
2	Horizontal target in the horizontal vs horizontal assay	Neutral box	7.72	<0.001
2	Horizontal target in the competitive assay	Neutral box	5.75	<0.001
2	Vertical target in the competitive assay	Neutral box	-2.77	0.048
2	Vertical target in the vertical vs vertical assay	Neutral box	-4.64	<0.001
3	Positive control target	Neutral box	-3.80	0.003
3	½ heated target	Neutral box	2.98	0.04
3	¼ heated target	Neutral box	2.91	0.048
3	⅛ heated target	Neutral box	3.58	0.01
3	Negative control	Neutral box	3.73	0.004

Table S2. Mean ± SEM densities per 100 cm2 of mosquitoes recovered on heated and unheated areas of targets, related to Figure 4.

Treatment	Mean ± SEM density of mosquitoes on the whole target	Mean ± SEM density of mosquitoes on the heated area	Mean ± SEM density of mosquitoes on the unheated area
Positive control	0.62 ± 0.08	0.62 ± 0.08	N/A
½ heated	0.56 ± 0.08	0.70 ± 0.12	0.42 ± 0.09
¼ heated	0.30 ± 0.09	0.63 ± 0.21	0.19 ± 0.06
⅛ heated	0.28 ± 0.05	0.80 ± 0.19	0.21 ± 0.05
Negative control	0.23 ± 0.03	N/A	0.23 ± 0.03

Table S3. Track parameters analysed with the custom made program, related to STAR Methods. Note that the notations "i" and "n" correspond to the initial data point and the final data point respectively of either the complete track or a segment of the track.

Parameter	Definition	Equation	Unit
3D linear distance	Shorter 3D distance between the first and last data point of a track, which is created by a straight line that joins the two points. Note the number is always positive.	$\sqrt{(x_n - x_i)^2 + (y_n - y_i)^2 + (z_n - z_i)^2}$	m
Total 3D distance (for an individual track)	Total 3D distance between the first and last data point of a track. This is calculated by adding all the 3D linear distances of all the consecutive points of a track, thus forming the 3D path of a given track.	$\sum_{k=i}^{n-1} \sqrt{(x_{k+1} - x_k)^2 + (y_{k+1} - y_k)^2 + (z_{k+1} - z_k)^2}$	m
Total track time (for an individual track)	Time between the first and last data point of a track or segment of a track.	$t_n - t_i$	s

Parameter	Definition	Equation	Unit
3D linear speed	Total 3D distance covered per time frame. This is calculated by dividing the total 3D distance by the total time elapsed between the points under consideration.	$\frac{\sum_{k=i}^{n-1}\sqrt{(x_{k+1}-x_k)^2+(y_{k+1}-y_k)^2+(z_{k+1}-z_k)^2}}{t_n-t_i}$	m s^{-1}
Tortuosity index	Index that indicates the convolutedness of a track. It is calculated as the ratio between the 3D linear distance and the total 3D distance. The values can vary from zero to one, where low values indicate a convoluted track (i.e. a 3D path that is longer compared to the shorter 3D distance between the two points), while values close to one indicate straighter tracks.	$\frac{\sqrt{(x_n-x_i)^2+(y_n-y_i)^2+(z_n-z_i)^2}}{\sum_{k=i}^{n-1}\sqrt{(x_{k+1}-x_k)^2+(y_{k+1}-y_k)^2+(z_{k+1}-z_k)^2}}$	N/A
